# Supplementary material for: Forecasting Staphylococcus aureus Infections Using Genome-Wide Association Studies, Machine Learning, and Transcriptomic Approaches
Source: mSystems. 2022 Jul 5;7(4):e00378-22. doi: 10.1128/msystems.00378-22 (PMC9426533; doi:10.1128/msystems.00378-22)
Supplement: FIG S1 [file msystems.00378-22-sf001.pdf]

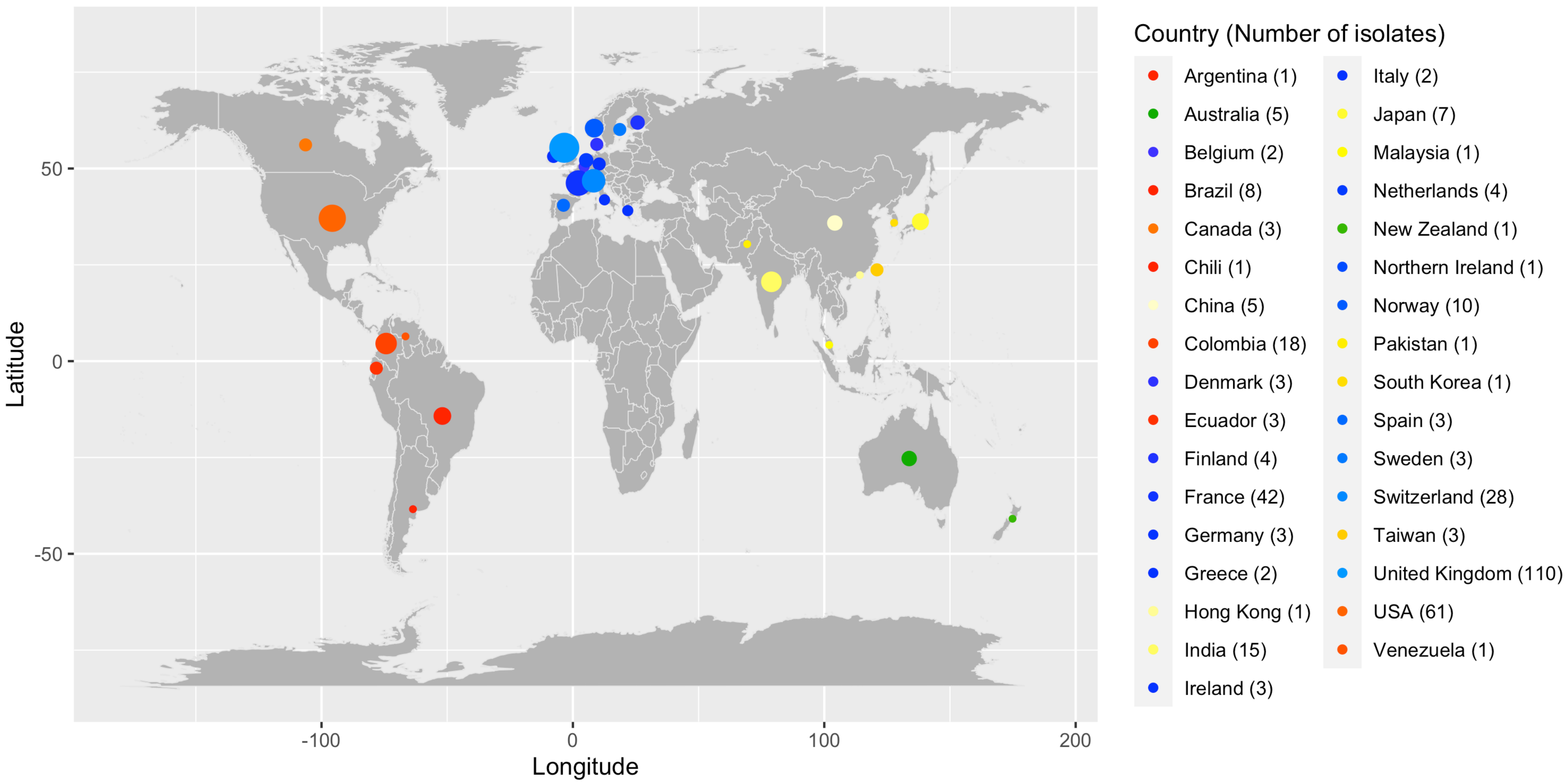
Country (Number of isolates)
Argentina (1)
Australia (5)
Belgium (2)
Brazil (8)
Canada (3)
Chili (1)
China (5)
Colombia (18)
Denmark (3)
Ecuador (3)
Finland (4)
France (42)
Germany (3)
Greece (2)
Hong Kong (1)
India (15)
Ireland (3)
Italy (2)
Japan (7)
Malaysia (1)
Netherlands (4)
New Zealand (1)
Northern Ireland (1)
Norway (10)
Pakistan (1)
South Korea (1)
Spain (3)
Sweden (3)
Switzerland (28)
Taiwan (3)
United Kingdom (110)
USA (61)
Venezuela (1)
Latitude
Longitude
50
0
-50
-100
100
200
