# Supplementary figures and images for: Forecasting Staphylococcus aureus Infections Using Genome-Wide Association Studies, Machine Learning, and Transcriptomic Approaches
Source: mSystems. 2022 Jul 5;7(4):e00378-22. doi: 10.1128/msystems.00378-22 (PMC9426533; doi:10.1128/msystems.00378-22)

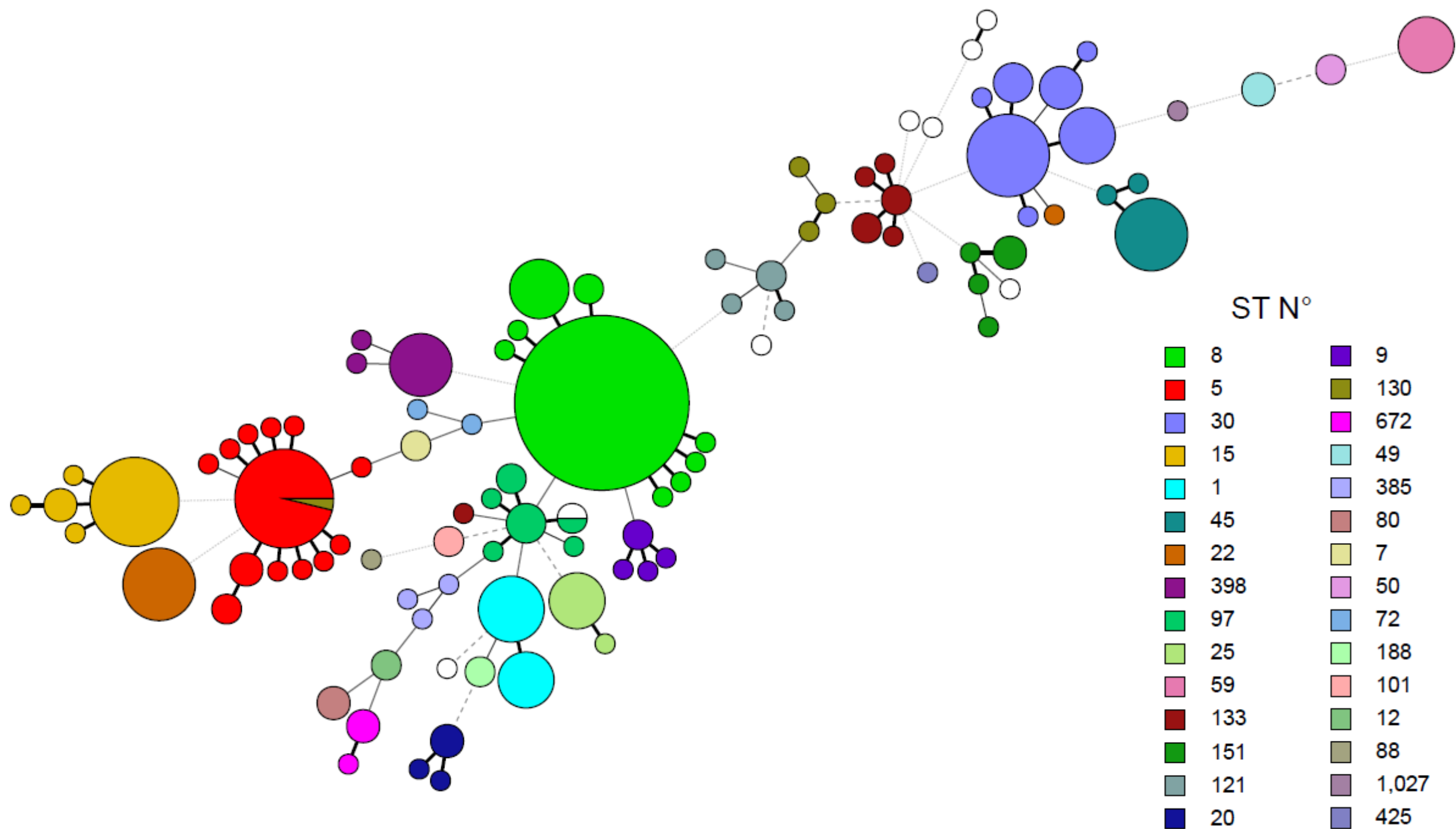

Supplement: FIG S2 [file msystems.00378-22-sf002.pdf]
